# Milk ejection patterns: an intra- individual comparison of breastfeeding and pumping

**DOI:** 10.1186/s12884-015-0583-3

**Published:** 2015-07-30

**Authors:** Hazel Gardner, Jacqueline C Kent, Ching Tat Lai, Leon R Mitoulas, Mark D Cregan, Peter E Hartmann, Donna T Geddes

**Affiliations:** School of Chemistry and Biochemistry, M310, The University of Western Australia, 35 Stirling Highway, Crawley, 6009 Western Australia; Medela AG, Lättichstrasse 4b, 6341 Baar, Switzerland

**Keywords:** Human lactation, Milk ejection, Breastfeeding, Milk expression, Breast physiology, Programming, Let down

## Abstract

**Background:**

Milk ejection is a transient episode critical to milk removal and women typically have multiple milk ejections during breastfeeding and pumping. Recently it was found that milk ejection characteristics such as number of milk ejections and periodicity were consistent throughout 12 months of lactation in women who expressed their milk with an electric breast pump. It is not known whether the stimulation of an infant at the breast influences milk ejection patterns or whether this is a programmed event. The aim of this study was to compare milk ejection patterns during breastfeeding and expressing milk with an electric pump within mothers.

**Methods:**

Twelve lactating mothers with normal milk production (502–1356 mL) had milk ejection recorded by measuring the diameter of a major milk duct with ultrasound imaging throughout an entire breastfeed and a 15-min pumping session. Scans were analysed for timing, duration of duct dilation and maximum duct diameter.

**Results:**

The initial milk ejection defined as the first increase in duct diameter was observed earlier during breastfeeding than during two phase pumping sessions but was not statistically significant (*p* = .057). There were no significant differences between the duration of the first or second milk ejection for mothers when breastfeeding or pumping at their maximum comfortable vacuum (*p* = .18; *p* = .99). The times taken to reach the peak duct diameter, or the first half of the milk ejection were also not found to be significantly different between breastfeeding and pumping.

**Conclusion:**

This study suggests that milk ejection patterns remain consistent within individual mothers regardless of whether the mother is breastfeeding or expressing milk indicating a likelihood of the process either being programmed or innate to the individual.

## Background

Breastmilk provides optimal nutrition and protection for infants. Breastfeeding is the most convenient method of milk removal, but expressing milk is important for many mothers, particularly in situations where the infant is premature, unwell, or where the mother is separated from her infant for extended periods of time. Stimulus of the breast differs markedly between breastfeeding and pumping and this is often offered as a potential explanation for the reduced effectiveness of the pump compared to the infant in some women [1]. In this study an electric pump was used but breastmilk may also be expressed by hand or using a manual pump.

Successful lactation is dependent on both milk synthesis and milk ejection. The alveoli in the human mammary gland are lined by lactocytes, which synthesise and secrete milk into the alveolar lumen. Myoepithelial cells that are comprised of smooth muscle fibres lacking neural innervation surround the alveoli. These myoepithelial cells contract in response to oxytocin that is released from the posterior pituitary gland into the blood stream in response to suckling or other stimuli [2, 3]. The contraction results in the expulsion of milk from the alveoli into the milk ducts [4]. Milk ejection in women is a transient phenomenon lasting 45 s to 3.5 min [5]. Unlike other species that possess cisterns such as the goat and cow [6, 7], the majority of milk in the human mammary gland is stored in the alveolar region [8] and requires active expulsion for successful lactation, such that the absence of the milk ejection reflex results in very little milk removed from the breast [1, 5].

Few non-invasive methods to detect and measure milk ejection exist. Ultrasound, however, has been shown to reliably and non-invasively detect milk ejection in lactating women both when breastfeeding and expressing milk with an electric breast pump [5, 8]. An increase in diameter of the milk ducts and movement of fat globules within the duct towards the nipple is indicative of milk ejection and occurs concurrently with an increase in milk flow rate during pumping [8, 9]. Duct diameter increases acutely during the first phase of the milk ejection, and decreases in the later phase.

Recently Prime and coworkers, in work from this laboratory, have shown that the timing, pattern and number of milk ejections are consistent in individual mothers during milk expression throughout the first 12 months of lactation [10]. It is not known whether this process is innately programmed or would differ in response to different stimuli. In this study we investigated whether individual milk ejection patterns remain consistent when mothers either breastfeed their infants or express milk with an electric pump.

## Methods

Twelve exclusively breastfeeding mothers provided written informed consent to participate in the study, which was approved by the Human Research Ethics Committee at the University of Western Australia. The studies were conducted in the research laboratory at the Breastfeeding Centre of WA at King Edward Memorial Hospital for Women.

The twelve lactating women measured their 24-h milk production in their own homes by test weighing their infants on accurate digital scales (BabyWeigh**™**, Medela Inc, McHenry IL, USA, resolution 2 g, accuracy ± 0.034 %) before and after each breastfeed from each breast for a period of twenty-four hours plus one breastfeed. The 24-h milk production was calculated by the method of Arthur et al. [11]. As no correction for infant insensible water loss was made, milk production may be underestimated by 10 % on average (range 3–55 %) [11].

During an initial visit to the Breastfeeding Centre, the maximum comfortable vacuum during pumping was ascertained for the left breast for each mother. An experimental electric breast pump (B2000, Medela AG, Baar, Switzerland), equipped with standard breast shield and bottle, was used. The pump was computer–driven, and the stimulation pattern (125 cycles/min) and expression pattern (54–78 cycles/min) were similar to those provided by the commercially available Medela Symphony breast pump (Medela AG, Baar, Switzerland). The vacuum level was adjustable (0–100 %) and the maximum applicable vacuum when the pump was set at 100 % was approximately −270 mm Hg. The breast shield was applied to the left breast, the pump was turned on to the stimulation pattern, and the vacuum was adjusted to the comfort of the mother. Following the detection of milk ejection (identified as milk duct dilation along with obvious milk flow towards the nipple), the pump was changed to expression pattern. Milk ejection was identified as milk duct dilation along with obvious milk flow towards the nipple as determined by ultrasound. The vacuum was then gradually increased further until the mother began to feel some discomfort. At this point the vacuum was reduced by 10 mm Hg, and this value was recorded as the maximum comfortable vacuum for that mother. Pumping continued at this vacuum strength for 15 min. The maximum comfortable vacuum was among a series of vacuums tested as reported by Kent et al. (2008) [12] and was found to result in optimum milk flow rate and milk yield in comparison with weaker vacuums.

A pumping session was conducted for each of the participants using the previously established maximum comfortable vacuum for each mother. A milk duct in the right breast was monitored with ultrasound using a linear array transducer (5–10 MHz) (Acuson XP10; Siemens, Mountain View, CA) and milk ejections were detected using the method described by Ramsay et al., (2004) [5]. In brief, a main duct was located in the lateral part of the breast not being pumped, close to the base of the nipple, and light pressure was applied to avoid compression or distortion of the duct. The scan plane spanned 40 mm from the nipple and interrogated to a depth of 30 mm. All ultrasound scans were videotaped for later analysis. Parker (Fairfield, NJ) Ultrasonic Gel was used for the scans.

The same mothers participated in at least one session where they breastfed their infants, during which a main milk duct in the contralateral breast was monitored by ultrasound as described above. Six of the mothers were scanned during one breastfeeding session, and six were scanned in multiple sessions: four mothers during two sessions, one mother during four sessions and one mother during five sessions. The majority of scans were on the left breast but in five of the sessions the right breast was monitored. The length of each breastfeeding session was dependent on the infant.

### Statistical analysis

Comparisons of the breastfeed and milk expression data were made from the first increase in duct diameter associated with the first milk ejection, with the duration of the data analysed determined by the duration of the breastfeed. The analyses were limited to the first two milk ejections. Changes in duct diameter were measured every 3 to 20 s during the breastfeeding and pumping sessions. This data was then plotted and used to determine the first increase in duct diameter, the time taken to reach peak duct diameter and the milk ejection duration. Milk ejection duration was calculated by measuring the time between the beginning of one milk ejection to the beginning of the next using the method described by Ramsay et al.[5]. Data are presented as mean ± standard deviation. *P* values <0.05 were considered statistically significant.

All analyses were performed using R version 2.15.0 for Mac OS X [13]. Additional packages were used for linear mixed modeling and lattice plots [14, 15]. The duct diameter was plotted from the initial increase in diameter and compared for both expressing milk using the maximum comfortable vacuum and for the infant feeding from the breast. The duration and time to reach the peak duct dilation during milk ejection were also measured.

Linear mixed effects analyses [15] of the relationship between milk ejection duration (for first and second milk ejections), time to peak (for first and second milk ejections) and the method of milk removal (breastfeeding or pump) were carried out with random effects of different intercepts for each mother.

## Results

Characteristics of the 12 exclusively breastfeeding mothers of healthy infants with normal growth parameters (no concerns expressed by the mother or the primary healthcare provider) are given in Table 1. The majority of the infants were male (n = 8) with six mothers being primiparous and six multiparous. There was no significant difference in relation to the time to first milk ejection associated with parity (*P* = .6).

**Table 1:** Participant characteristics

	Median	IQR
Mother		
Age (years)	33	31–35
Parity	2	1–2
Infant		
Gestational age at birth (weeks)	41	40–41
Current age (weeks)	18	15–19
Breastfeeding		
Number of feeds in 24 h	10	8–11
Total volume of breastmilk consumed in 24 h (mL)	874	773–980

The mean duration of breastfeeding sessions was 6 min 53 s (SD 2 min 57 s). The amount of milk consumed by infants during these sessions was variable with a mean volume of 82.7 mL (SD 47.3 mL).

The milk ejection duration and the time taken to reach peak duct diameter for breastfeeding were not significantly different between sessions for the six women observed during multiple breastfeeding sessions (*p* = .90; *p* = .84).

**Fig. 1:**
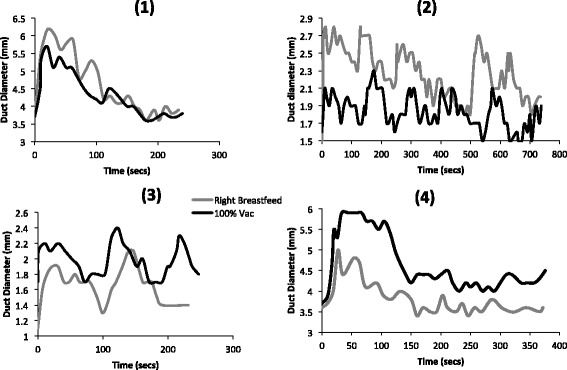
Milk ejection patterns in four individuals during breastfeeding and pumping with an electric breast pump at maximum comfortable vacuum. The pumping session was matched to the duration of the breastfeed for analysis.  Breastfeed  Pump at maximum comfortable vacuum

Representative examples of milk duct diameters during breastfeeding and pumping are shown in Fig. 1.

The milk ejection characteristics for the first two milk ejections are summarized in Table 2. The mean maximum comfortable vacuum was −201 ± 40 mm Hg (range −116 to −262 mmHg). Intra-oral vacuums applied by the infants during breastfeeding were not measured.

**Table 2:** The comparison of milk ejection characteristics within mothers when pumping and breastfeeding for milk ejections 1 and 2

	Milk ejection 1			Milk ejection 2		
	Breastfeeding	Pumping	*P* value	Breastfeeding	Pumping	*P* value
Duration (seconds)	105 ± 29	95 ± 27	.18	98 ± 17	98 ± 14	.99
Time to peak duct diameter (seconds)	34 ± 19	26 ± 16	.15	30 ± 13	40 ± 13	.16

There was a trend for the time to the first increase in duct diameter to be shorter when the infants were breastfeeding (53.6 ± 30.2 s) in comparison to pumping (73.3 ± 22.0 s; *p* = .057). The mean maximum duct diameter was similar for both groups (breastfeeding 3.45 ± 1.54 mm, pumping 3.44 ± 1.47 mm; *p* = .85).

There was no significant difference for individual mothers between breastfeeding and pumping in the duration of milk ejections (Table 2).

No significant differences were found when comparing the duration of the first milk ejection with subsequent milk ejections during pumping or breastfeeding sessions (*p* = .86).

The times taken to reach the peak duct diameter, or the first phase of the milk ejection were not significantly different for the first or second milk ejections during breastfeeding or pumping (Table 2). Furthermore, no significant difference was found between the time taken to reach peak duct diameter from the beginning of the first and subsequent milk ejections during breastfeeding or pumping sessions (*p* = .48).

## Discussion

In this study we used ultrasound to compare milk ejection characteristics between the two different stimuli - breastfeeding and pumping. The timing and duration of milk ejections for individual mothers were consistent during both repeated breastfeeding and pumping sessions suggesting that this is a programmed event regardless of the stimulus applied to the breast.

The time to the initial milk ejection during breastfeeding was on average 20 s earlier than during pumping sessions, however, this was not statistically significant and the result may have been different for a larger sample size. The breastfeeding infants initiated a milk ejection after 53 s in this study which is similar to a previous study which reported an increase in duct diameter 56 ± 30 s after the infant began to feed [5]. Similar results have been reported using other methods of measuring milk ejection during breastfeeding indicating that monitoring the duct diameter changes using ultrasound had not adversely affected time to milk ejection [16–18]. Kent et al. (2003) [1] found a significant difference in time to milk ejection between breastfeeding and in response to a range of stimulation patterns provided by an electric pump (120 – 149 s) suggesting there may be some variation in the timing of the release of oxytocin in response to different stimuli. This may have been due to the rigorous nature of that study during which the infant was in a separate room and silence was maintained between the mother and the researchers during the test sessions [1]. During a study under more relaxed conditions the time to milk ejection in response to a breast pump was 73–92 s [12]. These data and the current study, where the infant was present in the room, are consistent with a 20- to 30-s difference in time to milk ejection between breastfeeding and pumping. Whilst the breastfeeding infant tends to produce a more rapid initiation of milk ejection improving conditions during pumping may reduce the time to milk ejection.

When comparing milk ejection characteristics between breastfeeding and pumping we found that the periodicity (timing) and duration of the milk ejection did not differ between breastfeeding and pumping within a mother. There were no significant differences between the duration in either the first or second milk ejections when mothers were breastfeeding or pumping (Table 2). The analysis was limited to two milk ejections as the monitored breastfeed was typically much shorter (6 min 53 s ± 2 min 57 s) than the pumping session of 15 min but the duration of milk ejections was comparable to findings described by others [5, 10]. Further, we found that the time taken to reach the peak duct diameter for the first and second milk ejections to be no different for breastfeeding and pumping. These results are similar to those of the pumping studies conducted by Prime et al. in that milk ejection patterns during breastfeeding appear to remain consistent during exclusive breastfeeding in the short term, (over a six week period) however longitudinal studies are necessary to confirm if they remain constant for the full length of lactation [10].

The vacuum characteristics of breast pumps in general differ to that of the breastfeeding infant. The infant typically displays a higher suck cycle rate (74 sucks/min during nutritive sucking [19]) compared to the cycle rate of the pump used in this study (ranging between 54 and 78 cycles/min depending on the set level of vacuum [20]). However, once set, the vacuum strength and the cycle rate were maintained for the full expression session. In addition, the infant not only sucks in bursts with intervening pauses to ensure maintenance of cardiorespiratory stability [21], but also varies the strength of vacuum within a suck burst and typically uses either the same or stronger vacuums during non-nutritive sucking. Although intra-oral vacuums were not measured in this study, published figures are −148 ± 58 mm Hg, which is lower than the pumping vacuum used in this study. Despite these differences in strength and frequency of application of vacuum between breastfeeding and pumping, the measured milk ejection characteristics were similar. Pumping patterns using more variation to more closely resemble infant feeding have also shown no effect on milk ejection characteristics other than to have a shorter time to the first milk ejection [8]. Interestingly we have previously found that different vacuum strengths during pumping do not affect the number of milk ejections within a mother [12]. We conclude that, once stimulated, the milk ejection pattern is robust and independent of strength and frequency of application of vacuum.

In this study we found for the first time that the duration of multiple milk ejections within either a breastfeed or pumping session to be similar within women. This indicates that the amount of oxytocin released at each milk ejection is similar [16, 18] irrespective of the mode of stimulation and milk removal. Indeed it has been reported that there is no significant difference in oxytocin levels when comparing women breastfeeding with expressing milk using a variety of mechanical methods [22]. Stress is also known to affect the milk removal seemingly via the milk ejection process through negatively influencing oxytocin release and impaired milk ejection [23]. Stress was most likely minimized during pumping in this study as the mothers attended an orientation session before the session that was analysed for this paper, which was randomized amongst 3–4 different vacuum conditions [12]. It is unlikely therefore that we would detect changes in milk ejection patterns as a result of stress. Further study would be required to confirm if indeed the timing and duration of milk ejections are actually affected by stress.

Although milk ejection patterns are similar in women when breastfeeding or pumping, a significant portion of women whose infants are able to remove substantial amounts of milk are unable to pump milk effectively [12]. Further, the number of milk ejections during breastfeeding is positively related to the volume of milk removed by the infant [5], however, this has not been confirmed during pumping. These data suggest that factors other than milk ejection characteristics are influencing the effectiveness of the pump in removing milk and warrant further investigation.

Not only have we demonstrated that milk ejection patterns are similar between breastfeeding and pumping but that multiple breastfeeding sessions for individual women show consistent milk ejection patterns, at least over a period of 4–5 weeks. These results indicate that milk ejection characteristics are similar in the short term; however, monitoring over twelve months is necessary to confirm that these remain consistent in the long term.

Infant survival is dependent on the ability to feed effectively, and it is acknowledged that gestation and early infancy are critical periods for programming feeding. Disruptions during this period such as premature birth and caesarian section are amongst events known to impact feeding outcomes [19, 24]. The nature of milk ejection must therefore be robust to cope with insults to the infant; however, the infant’s role if any in programming maternal milk ejection patterns is unknown. Recent animal studies suggest that programming in relation to lactation performance and offspring milk intake may begin well before birth [25, 26], however, the timing of the programming of the milk ejection reflex or even if such programming occurs remains unclear.

It is well documented that infants regulate their intake of breastmilk according to their appetite [27] and display individual characteristics in relation to the rate of milk transfer and duration of feeding [28]. Whether these variations are infant determined, according to appetite, or are somewhat dictated by milk ejection patterns remains to be determined. These results appear to indicate that the milk ejection pattern for the mother is innate, further research is required to investigate whether this pattern is influenced by the initial stimulation by the breastfeeding infant immediately postpartum or if it is programmed during pregnancy.

The limitations of the study include the relatively small sample size. In addition, this study only uses one type of electric pump so therefore does not presume to suggest that these results would be replicated with the use of other electric pumps, manual pumps or indeed expressing milk by hand. Additional research is required to ascertain if milk ejection patterns remain consistent during other methods of milk removal.

## Conclusion

This study found that milk ejection patterns are not different within individual mothers when breastfeeding or expressing milk with an electric pump set at maximum comfortable vacuum. We also found that milk ejection patterns remain consistent during breastfeeding sessions monitored over several weeks. Both of these results support the possibility that milk ejection is an innately programmed physiological response. This implies that factors other than milk ejection characteristics play a role in determining the amount of milk removed by the breast pump.
